# A New Method to Make 24-Hour Urine Collection More Convenient: A Validity Study

**DOI:** 10.1155/2014/718147

**Published:** 2014-05-20

**Authors:** Pooneh Nabavizadeh, Shadi Ghadermarzi, Mohammad Fakhri

**Affiliations:** School of Medicine, Shahid Beheshti University of Medical Science, Tehran, Iran

## Abstract

*Background and Objectives*. This study proposes a novel urine collection device that can divide each urine collection into 20 parts and store and cool just one part. The aim of the current study is to compare measured biomarkers from the proposed urine collection device to those of conventional 24-hour sampling method. We also hypothesized that the new method would significantly increase patients' adherence to the timed urine collection. *Methods*. Two 24-hour urine samples with the conventional method and with the new automated urine collection device that uses just one-twentieth of each void were obtained from 40 healthy volunteers. Urine parameters including volume, creatinine, and protein levels were compared between the two methods and the agreement of two measurements for each subject was reported through Bland-Altman plots. *Results*. Our results confirmed that for all three variables, there is a positive correlation (*P* < 0.001) between the two measurements and high degree of agreement could be seen in Bland-Altman plots. Moreover, more subjects reported the new method as “more convenient” for 24-hour urine collection. *Conclusions*. Our results clearly indicate that a fixed proportion of each void may significantly reduce the urine volume in timed collections and this, in turn, may increase subjects' adherence to this difficult sampling.

## 1. Introduction

Urine samples are one of the most frequently used and invaluable specimens in current medicine. There are several biomarkers that help final diagnosis in many nephrologic and endocrine abnormalities. Timed specimens may be essential for quantitative measurement of certain biomarkers, comprising those with diurnal variation. Of these variables, urine protein plays a fundamental role in the evaluation of patients with renal diseases and may also foresee those who will develop end stage renal disease [1, 2]. Thus far, 24-hour urine collection was the standard method to quantify proteinuria but inconvenience and inaccuracy due to possible errors in sample collection lead to new substitutions such as spot urine sampling for this difficult method. This is while for many other biomarkers requiring timed specimens such as creatinine or analytes such as endocrine measures including catecholamines and 17-hydroxysteroids, no other convenient method is available.

Indeed, difficulty and inconveniency of the 24-hour sampling method, along with certain errors in collection of the specimen, have confined the usefulness of this sampling method. Errors such as loss of specimen from poorly sealed container and improper storage of the urine throughout the day are two main obstacles with this method of sampling [3, 4].

Urine specimen should be kept in a cool place during collection to lower false results as a result of enzymatic reactions, which depend on temperature. This usually requires use of home's refrigerator that may significantly lower patient's adherence to standard storage guidelines.

To overcome the abovementioned limitations of current 24-hour sampling method, this study proposes a novel urine collection device that can divide each urine collection into 20 parts and store and cool just one part. This automated device will cut the final volume of the sample to one-twentieth and no refrigerator is needed. We hypothesized that sample's quality and measured biomarkers from the proposed urine collection device are comparable to those of current 24-hour sampling method but may significantly increase patients' adherence and satisfaction.

## 2. Methods

### 2.1. Patients

A total of 40 healthy volunteers were enrolled in this study. Inclusion criteria of this study required all subjects to be between 18 and 40 years of age with no positive history of any nephrologic and/or endocrine diseases. Exclusion criteria included pregnancy, history of hypertension, and use of substances interfering with creatinine excretion such as commonly prescribed H2 blockers, antibiotics with renal excretion, and protein supplements. All study participants were fully informed by the primary investigator about the aims of the study and an informed consent was obtained from all cases. The entire study protocol, as well as a detailed description of the proposed device, was reviewed and approved by medical ethics committee of Kurdistan University of Medical Sciences.

### 2.2. New Collection Device Specifications

The automated urine collection device used in the current study is an invented tool, which was developed solely for purpose of timed urine collections. This device is registered as a national invention in Iran (reference number 81571). Using this portable apparatus, urine samples from each voiding session will be automatically divided into twenty equal and independent portions. The device encompasses a generally vertical manifold having an upper input and a landslide of outputs at the lower end to depose 19 portions and keep just one portion.

Since urinary concentration changes with each sample voided, it is essential to distinguish the typical concentration over a given period of time. This device warrants an identical size of each accumulated specimen to stream into each section unaffected, thus establishing alike urinary chemistry compositions.

**Figure 1:**
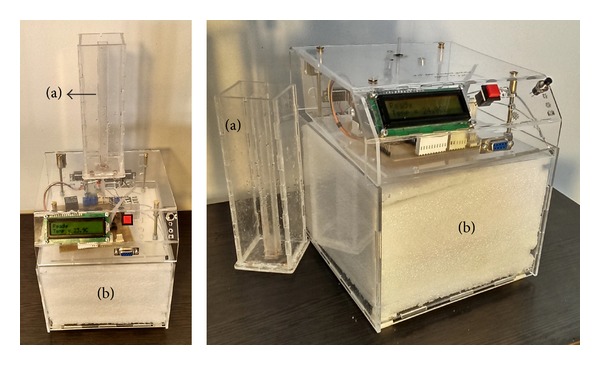
Automated urine collection device. (a) Disposable container. (b) Main body.

As illustrated in Figure 1 the device is made up of a disposable container and a main body. The disposable container in which the patient urinates is 5 × 4 × 35 cm and the main body of device is 30 × 30 × 40 cm. The main body consists of a removable receptacle and a cooling system. The disposable container has an inside bin, the cross section of which is one-twentieth of the whole container. The container is adapted to fit on the main body of the device and is to firmly engage it. The inside bin has an opening designed to register with the inlet of the receptacle placed inside the main body. The urine inside the receptacle is preserved for 24 hours in favorable temperature (below 5 degrees of Celsius) provided by an electronic cooler. There is also a digital display on the device indicating inside temperature and the device status.

**Figure 2:**
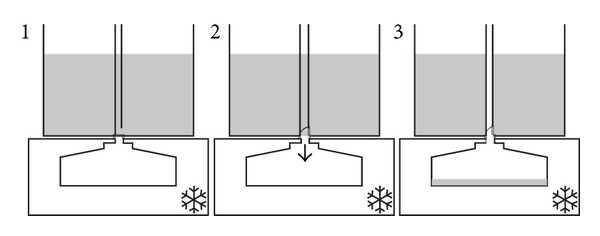
Schematic view of the device process. Step 1. A fifteen-second pause to ensure that there is no turbulence in the liquid during which the inside bin and the container are connected while the receptacle and inside bin are not. Step 2. Disconnection of the inside bin and the container resulting in separation of one-twentieth of the sample. Step 3. Connection of the inside bin and the receptacle. The device will stay in this phase for 15 seconds allowing complete discharge of the inside bin content to the receptacle.

After voiding in the container, it is placed on the main body of device by the patient. Pushing the start button the device automatically passes three steps as shown schematically in Figure 2.

### 2.3. Data Collection

All study participants were provided a conventional 24-hour urine container as well as the new automated urine collection device. A written description of the new device was handed to the participants. Meanwhile, verbal instructions to help participants with their collections were provided.

For each study participant, two separate urine collections were obtained in two consecutive days. The first sample was taken with conventional 24-hour urine collection instructions. In the second day, another sampling was performed using the new automated urine collection device. For the first sampling (conventional collection), participants were asked to start urine collection at any time in the morning, after discarding first morning urine. After that, entire volume of urine throughout the day should be collected in the conventional container that was handed to all subjects. The same technique (disposal of the first void) was recruited for the new sampling device and participants were just asked to void into the automated device for the second day collection. Subjects were strictly advised to keep their fluid intake the same in both days.

To determine adequacy and test validity of urine collection with the new method, three main variables were studied. Urine creatinine, volume, and protein levels were compared between the two methods of collection. Urinary creatinine concentrations were reported by the kinetic Jaffé method utilizing a Roche modular analyzer. Urine protein levels were determined with the turbidimetric method with the aid of benzethonium chloride.

Since the major drawback of the conventional 24-hour sampling method is low patients' adherence to instructions, we also measured level of appropriateness and subjects' satisfaction and comfort when collecting 24-hour urine with each method through a self-report measure with 5 scales (1 = complete dissatisfaction, 5 = easiest and most appropriate).

### 2.4. Statistical Analysis

Continuous variables including volume of the urine, creatinine, and protein for either method were presented as mean ± SD. Moreover, categorical variable (patients' satisfaction) was presented with frequency and percentages. Since the observations for the continuous variables were related for each subject, nonparametric analysis with Wilcoxon signed-rank test was utilized to show possible difference between the recordings of the two methods of urine collection. Moreover, chi-square test was used to report possible difference between patients' satisfaction towards each method. Pearson correlation was used to depict possible correspondence between the two methods. The two measurements for urine creatinine and protein by both methods were also compared by Bland-Altman plot. Bland-Altman analysis shows the degree of agreement when linear correlation is unsuitable because of relation of the two recordings [5, 6]. To do this, the difference between estimated and actual measurements of both protein and creatinine was plotted against the average of the measurements. Limits of agreement were also showed to allow interpretation (mean of difference between the two measurements ± 2∗SD). A *P* value < 0.05 was considered to be significant in this study. All statistical analyses were performed using SPSS version 20.0 for Mac OS.

## 3. Results

A total of 40 healthy subjects with mean age of 28 ± 18 were enrolled in the study. One sample was excluded because the volume of both measurements was more than 3000 cc. Of the remaining cases 22 were female (56.4%) and 17 were male (43.6%). Our results showed that urine volume from the conventional 24-hour sampling method was 1532 ± 355 cc, while the adjusted volume (multiplied by 20 as the automated collection device keeps one-twentieth of the urine in each void) for the new sampling was 1510 ± 361 cc. Wilcoxon signed-rank test showed no significant difference between the two methods (*P* = 0.61).

**Figure 3:**
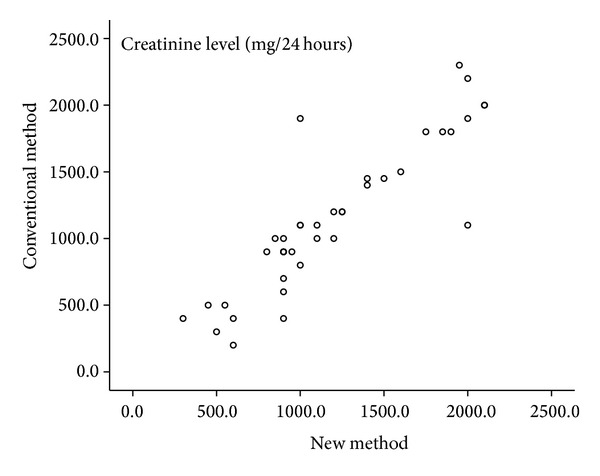
Conventional plot: Urine creatinine levels in 24 hours as measured by conventional and new methods.

**Figure 4:**
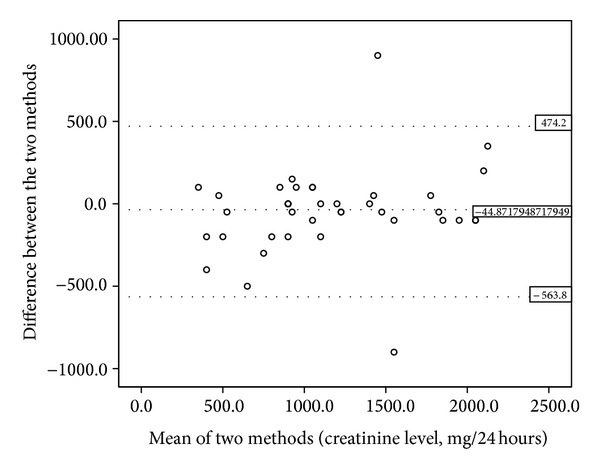
Bland-Altman plot: Relation between the new method and the conventional 24-hour urine creatinine. The horizontal lines show the limits of agreement of the differences between the two methods.

For the urine creatinine levels, the mean value for the conventional method for all subjects showed to be 1148.7 ± 558.0 mg/24 hours whereas this value from the new sampling method (adjusted by multiplying by 20) was 1193.6 ± 508.0 mg/24 hours. This variable also showed no statistically significant difference between the two methods (Wilcoxon's *P* = 0.13). Detailed demonstration of the two measurements was plotted in Figures 3 and 4 (conventional and Bland-Altman plots). The horizontal lines in Figure 4 show the limits of agreement of the differences between the old sampling method and the new method. Pearson correlation revealed a statistically significant correlation between the two methods (*P* < 0.0001, *r* = 0.79). Since this value may be different between men and women, further analysis in gender groups for the conventional method showed a mean value of 1338.6 ± 601.5 and 902.9 ± 388.3 mg/24 hours for women and men, respectively. The adjusted amounts of urine creatinine as measured from the second sampling (new method) were 1327.3 ± 527.3 and 1020.6 ± 438.0 mg/24 hours, respectively. Separate paired analysis of each gender was also failed to show a statistically significant difference between the two methods (*P* = 0.09).

**Figure 5:**
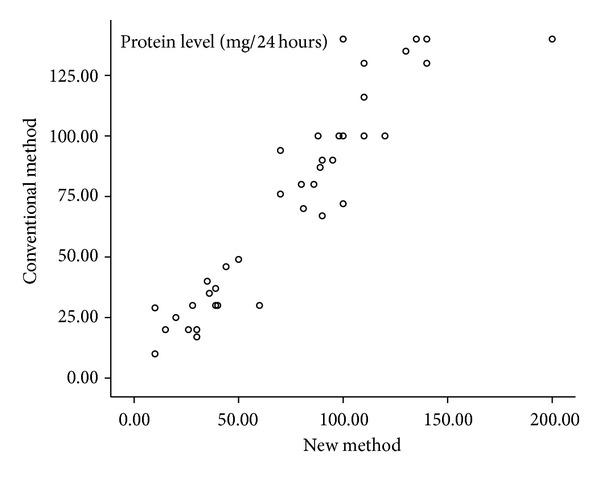
Conventional plot: Urine protein levels in 24 hours as measured by conventional and new methods.

**Figure 6:**
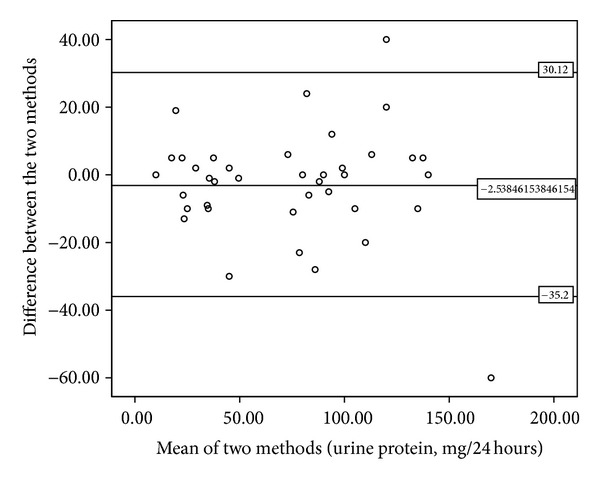
Bland-Altman plot: Relation between the new method and the conventional 24-hour urine protein. The horizontal lines show the limits of agreement of the differences between the two methods.

This study also compared the measured protein level between the conventional 24-hour sampling method and the new automated collection to see possible difference. Our results showed that in the conventional method the amount of 24-hour protein in the urine is 72.95 ± 42.13 mg/24 hours. The adjusted number (multiplied by 20) amount for the new collection method was 75.49 ± 43.74 mg/24 hours. There was no statistically significant difference between the two levels (Wilcoxon's *P* = 0.32). As shown in Figure 5, a positive correlation is present between the two methods regarding the protein levels (Pearson *r* = 0.93, *P* < 0.001).  Figure 6 also shows the relation of two protein measurements by the Bland-Altman plot.

Subjects' satisfaction with each collection method was showed to be very different. Our data revealed that all of 39 subjects have selected a higher scale (more satisfaction) with the new method. These results are summarized in Table 1. Chi-square test showed a statistically significant difference between the two methods with regard to participants' satisfaction (*P* < 0.05).

## 4. Discussion

This study proposed a more convenient method for 24-hour urine collection. Results of this study clearly indicate that storing a predefined and small proportion of each void not only facilitates the collection and makes the final 24-hour urine sample smaller, but also preserves its diagnostic abilities. Indeed, our preliminary results with the new urine collection device are comparable to those of previous conventional timed collection method.

Thus far it has been stated that the difficulties associated with using 24-hour urine collections could be identified in approximately one-third of subjects [7]. Cumbersome nature of conventional 24-hour urine sampling along with its technical difficulties may result in inaccurate sampling. Moreover, cultural stigmata of carrying large bottle of urine during the collection may result in significant decrease in adherence of patients to do this sampling method [8]. Undeniably, the large volume of sample is one of the major reasons for patients' refusal of the conventional sampling method. To address this shortcoming of the sampling, spot urine samples showed to be as accurate as timed urine collections for many variables including calculations of variety of indicators of kidney function [9–11]. But this compromise of timed sampling is not applicable for some clinical settings and 24-hour urine collection is still mandatory. In fact in clinical settings where patients have unusual body habitus including obesity and amputations, for those who need chemotherapy regimens, and during evaluations of potential kidney donors, timed urine collections are still standard method of urine collection and evaluation [12–14].

Rather than using spot sampling, our study came up with a novel approach that meaningfully ease the process of 24-hour sampling but also maintains its accuracy. Our results have showed that the three main indicators of proper sampling including volume, urine creatinine, and urine protein as measured by our proposed method are all in agreement with the conventional technique. Indeed, dividing each void into 20 parts and storage of one portion will significantly reduce the specimen's size and facilitate the collection and transport of the sample while maintaining its diagnostic value. Moreover, this new method has significantly increased subjects' satisfaction with the sampling method. This may help increase patients' adherence to this difficult urine collection method and reduce the need for repeating measurements due to improper collections.

This study suffers from certain limitations. Rather small sample size and confining the study participants to normal and healthy volunteers may preserve the internal validity but decrease the extensibility of our results. Moreover, in the current study, we just focused on three main variables of the urine: volume, creatinine, and protein levels. Further studies with more biochemical measurements may help validate our preliminary results and show possible success and applicability of this new collection device in many endocrine and nephrologic diseases. We recommend new studies with the proposed device on patient population with larger sample size to increase the external validity of the study for more urinary variables. Moreover, using detailed and standardized questionnaires with multiple items measuring patients' satisfaction may be more proper as compared to our rudimentary five-scale measurement.

## Figures and Tables

**Table 1:** Satisfaction of patients with each 24-hour collection method.

Satisfaction scale	1 (least)	2	3	4	5 (most)
Conventional collection method	28 (71.8%)	7 (17.9%)	2 (5.1%)	1 (2.6%)	1 (2.6%)
New collection method	1 (2.6%)	2 (5.1%)	12 (30.8)	21 (53.8%)	3 (7.7%)
